# Fish use of deep-sea sponge habitats evidenced by long-term high-resolution monitoring

**DOI:** 10.1038/s41598-025-01822-5

**Published:** 2025-05-21

**Authors:** Laurence H. De Clippele, Claude Nozères, Jinshan Xu, Barry MacDonald, Camille Lirette, Kirk Phelan, Calisa Staniforth, Fred Whoriskey, George A. Wolff, Sabena Blackbird, Christian Mohn, Ellen Kenchington

**Affiliations:** 1 School of Biodiversity, One Health and Veterinary Medicine, University of Glasgow, Glasgow, G12 8QQ UK; 2 Department of Fisheries and Oceans, Bedford Institute of Oceanography, Dartmouth, NS B2Y4A2 Canada; 3 Ocean Tracking Network, Dalhousie University, Halifax, NS B3H 4R2 Canada; 4 School of Environmental Sciences, University of Liverpool, Liverpool, L69 3GP UK; 5 Department of Ecoscience, Aarhus University, 4000 Roskilde, Denmark

**Keywords:** Sponge grounds, Habitat use, Fish, Behaviour, Seasonal, Diel vertical migration, Behavioural ecology, Biodiversity, Ecosystem ecology, Ocean sciences

## Abstract

It is critical that fish’s habitat uses of benthic habitats are understood, to inform effective fisheries management and to predict the impacts of human activities and climate change. In this study, benthic landers were used to collect long-term high-temporal resolution data to gain insights into the habitat use of sponge grounds by fish at the Sambro Bank Conservation Area. An integrated ecosystem-based monitoring approach was used, involving data collected on the biology, food supply, and oceanography. Fish abundance, behaviour and complex benthopelagic interactions were analysed over spatial and extended temporal scales (i.e., 30-min intervals from 2021 to 2023). A total of 21 different planktivorous and benthivorous fish taxa were found to utilise the seafloor. We show that sponge grounds can act as a nursery, feeding and shelter habitats for commercially important fish. In-depth analyses of Redfish, urophycid hakes, and Silver Hake revealed distinct diel and seasonal patterns and showed how food, sponge density and current speed are important drivers of their abundance and behaviour. Supported by fishery trawl survey reports, high-temporal resolution benthic ecosystem monitoring revealed the importance of sponge grounds and environmental drivers to commercially important fish. Such information is crucial for developing and implementing robust, evidence-based policy and management.

## Introduction

Understanding the nature and extent of a species’ habitat use is important for predicting the impacts of human activities and climate change on the distribution, reproduction and survival rates of the species^1–3^. In coastal marine ecosystems, fish use a variety of benthic habitats for feeding, shelter and nurseries^4–6^. However, few studies^7–14^ have investigated the habitat use by fish in offshore waters exceeding 100 m in depth.

Gaining insights into habitat use by fish in such locations is challenging. Acoustic methods (e.g., echosounders), which can be used to assess presence and biomass for fish stock assessments, are of limited utility near the seabed as fish in the “Acoustic Dead Zone” cannot be resolved from seabed echoes, which only visualise those with swim bladders^15,16^. Samples taken as part of trawl surveys and visual observations collected by submersibles, remotely operated vehicles (ROVs) or autonomous operated vehicles (AUV) provide data for points in time^11,17,18^. These approaches are not suitable for tracking changes at a high temporal frequency (e.g. every hour) over a long period (e.g. a year), hence, changes in the abundance or behaviour of species at diel and seasonal scales often remain unknown or understudied, unless long-term image observations are conducted^19^. Moreover, not only is trawling destructive to sponge habitats^20^ it also only provides low spatial resolution data (at the km-long scale), whereas the noise and light emitted by trawling, ROVs and AUVs can interfere with fish behaviour^21,22^. Furthermore, cryptic and smaller-sized fish may be poorly sampled in trawl catches. Consequently, our understanding of the nature and strength of deep offshore habitat-fish associations remains poorly resolved and can be biased by the chosen survey method.

Understanding the use of offshore and deep (> 100 m) habitats by fish at spatial scales relevant to individuals requires prolonged in-situ observations at high temporal and spatial resolutions. Benthic landers, stand-alone stationary seafloor observation platforms that can be deployed for extended periods^23,24^, can deliver datasets covering longer temporal periods and depending on their configuration, a variety of environmental covariates. They have successfully supported studies of fish behaviour although with the trade-off that they cover only one point in space^13,24,25^.

Sponge grounds, which are found globally, are vulnerable habitats that are important because of their role in nutrient recycling^26–31^ and because they act as habitats for a rich diversity of fish and benthic organisms^32–38^. However, to date, there is very limited evidence on the habitat use of sponge grounds by fish.

Data collected via trawl surveys show that high fish diversity and biomass can be found in sponge grounds^8,12^. In the deeper waters of the northwest Atlantic continental shelves, several deepwater species, such as the Sawtooth eel (*Serrivomer beanii*), deep-sea catshark (*Apristurus profundorum*), eelpouts (*Lycodes* spp.) and Acadian Redfish (*Sebastes fasciatus*), have strong positive associations with sponge biomass at the scale of individual trawl tows^8,39^. While a different study conducted on the Canadian shelf indicated that the mean abundance of Redfish was higher outside sponge grounds, it noted that glass sponges formed by species such as *V. pourtalesii* (Class Hexactinellida) provide a unique niche for Redfish as they are sometimes observed inside the barrel-shaped sponge itself^35^. In the same area, it has also been demonstrated that these sponges are used as shelters for Redfish in high currents^13^. While understudied, a few studies have shown that certain deep-sea fish species eat sponges and/or sponge remains^40,41^. Although the literature suggests that sponges and sponge grounds play important roles as a habitat for a wide range of organisms, the strength of the associations and their ecological linkages remains understudied^8^.

**Fig. 1:**
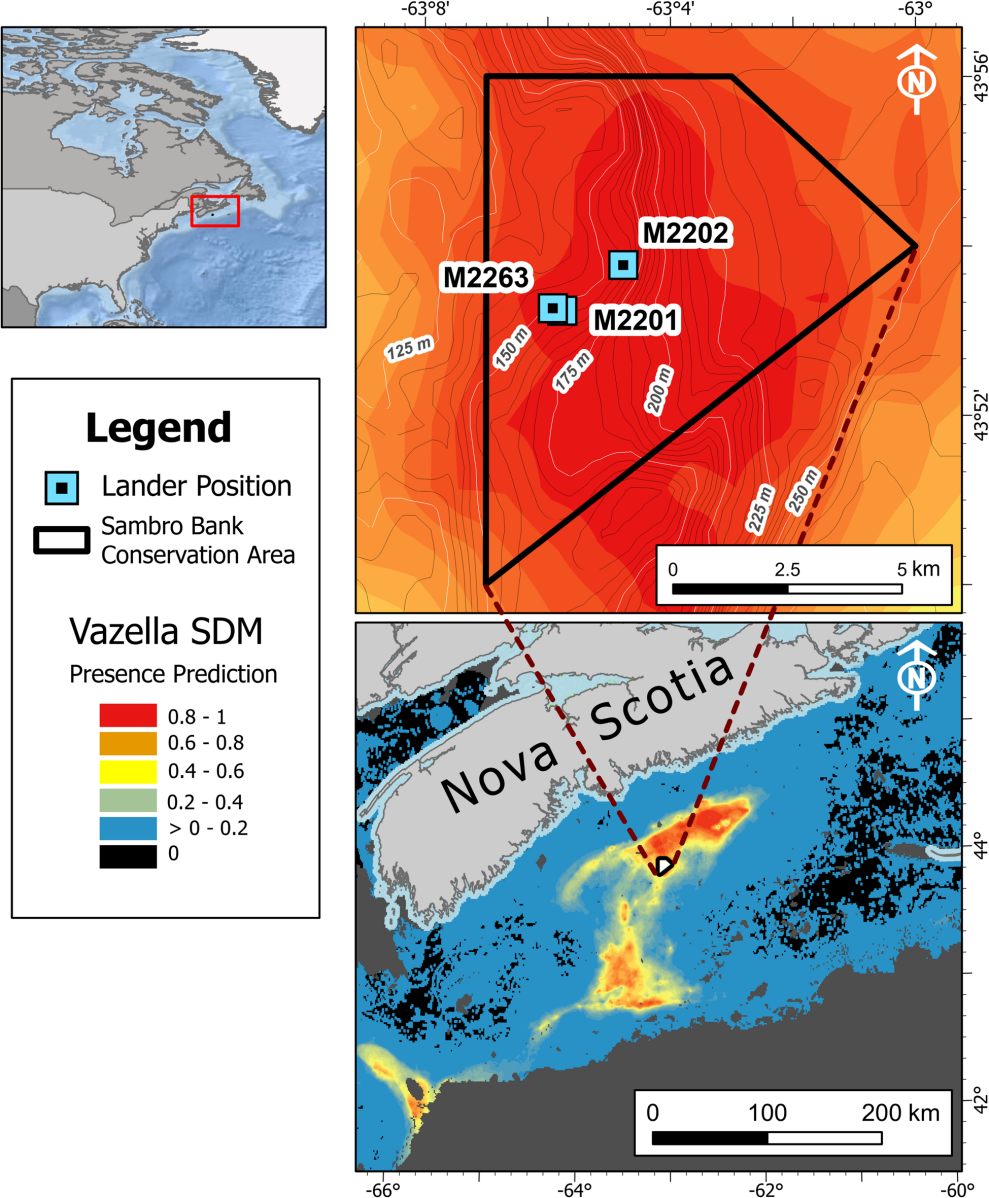
Study Area: Map showing the modelled presence of the species *Vazella pourtalesii* (Beazley et al. 2018), the deployment locations of the benthic landers, and the extent of the Sambro Bank Conservation Area. Lander M2201 and M2202 were deployed in 2021–2022, and lander M2263 was deployed in 2022–2023.

This study aimed at understanding the habitat use of offshore sponge grounds in the Sambro Bank Conservation Area, located on the Scotian Shelf, in the northwest Atlantic (Fig. 1), particularly their use by Redfish, urophycid hakes (*Urophycis tenuis* and *U*. *chuss*) and Silver Hake (*Merluccius bilinearis*). Each of these species are frequently observed on the seabed in the conservation area. The Sambro Bank conservation area extends an area of 62 km^2^, and ranges from 150 to 175 m depth, within which all commercial bottom-contact fishing gear is prohibited. The Scotian Shelf sponge grounds, characterised by dense aggregations of the glass sponge *Vazella pourtalesii*, are far more extensive than the conservation area, covering ~ 8000 km^2^ at depths of^41^ 87 to 498 m^42–44^. The sponges can be relatively large, up to 110 cm in height^45^, and sustain a high diversity of epifauna^35,42,46^. To study fish habitat use, we adopted an integrated monitoring approach. Our primary data source was high-resolution time-lapse seabed imagery collected over extended periods using benthic landers.

## Results

### Fish presence and behaviour

**Fig. 2:**
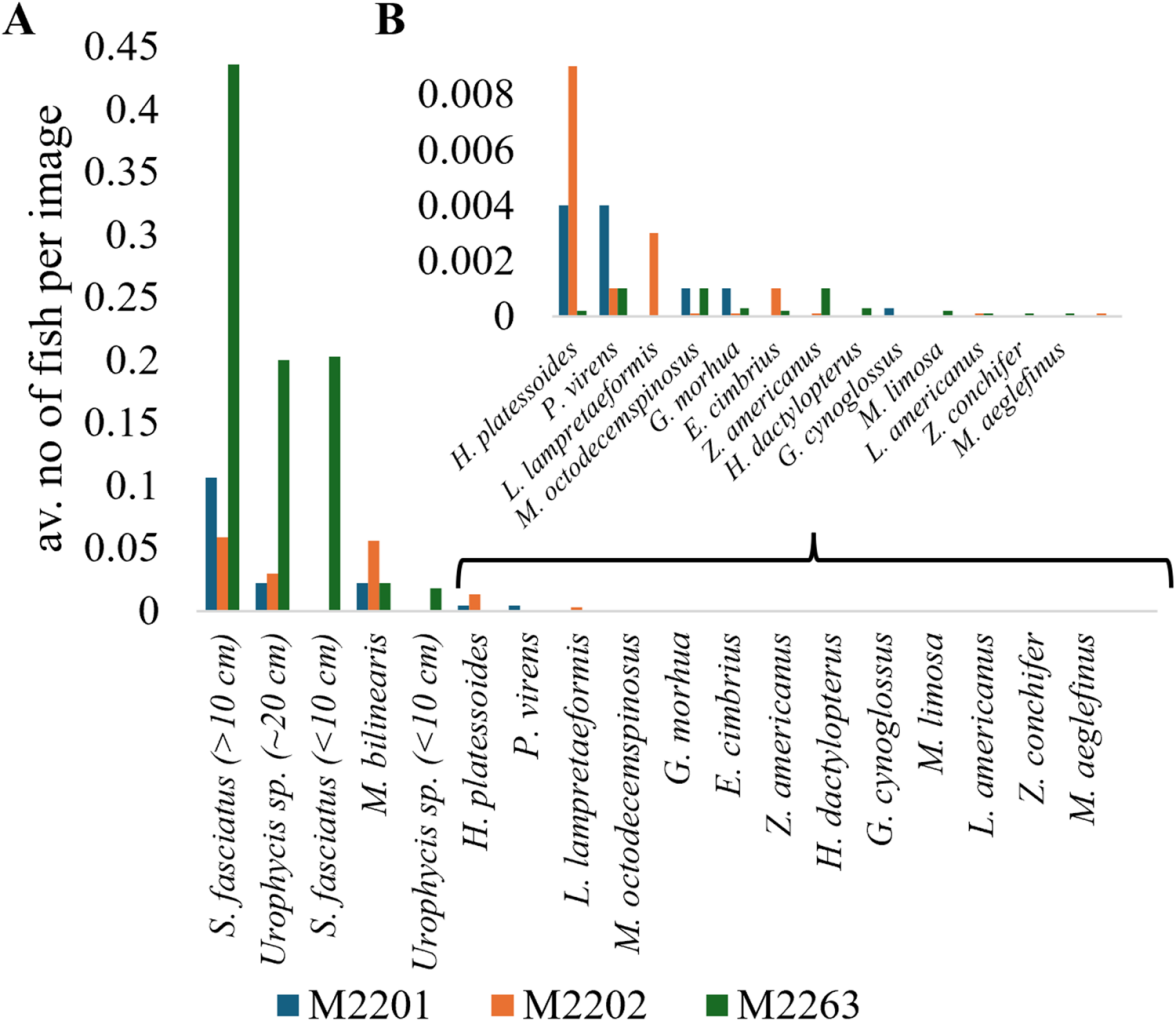
Fish records: Average number of fish recorded in the time-lapse images, by lander deployment. The absolute and average abundances and their standard deviations are provided in the Supplementary Materials. Fish common and Latin names: Redfish (*Sebastes fasciatus*), Silver Hake (*Merluccius bilinearis*), American Plaice (*Hippoglossoides platessoides*), Pollock (*Pollachius virens*), Snakeblenny (*Lumpenus lampretaeformis*), Longhorn Sculpin (*Myoxocephalus octodecemspinosus*), Atlantic Cod (*Gadus morhua*), Fourbeard Rockling (*Enchelyopus cimbrius*), Ocean Pout (*Zoarces americanus*), Blackbelly Rosefish (*Helicolenus dactylopterus*), Witch Flounder (*Glyptocephalus cynoglossus*), Girard’s Hagfish (*Myxine limosa*), Monkfish (*Lophius americanus*), Buckler Dory (*Zenopsis conchifer*), Haddock (*Melanogrammus aeglefinus*) and Striped Atlantic Wolffish (*Anarhichas lupus*).

A total of 21 fish species were recorded as part of the three landers, which were deployed with time-lapse cameras and Ocean Tracking Network (OTN) receivers (Fig. 2, Supplementary Tables 1–3).

As part of lander M2201 (deployed 2021–2022), 2793 images, spanning a period of ~ 58 days were recovered. In contrast, lander M2202 (deployed 2021–2022) captured 11,799 images over 245.8 days and lander M2263 (deployed 2022–2023) yielded 11,794 images taken over 245.7 days^47^. A total of 17 different species were recorded in the images. The most common species were Redfish (*Sebastes fasciatus*), followed by urophycid hake, Silver Hake (*Merluccius bilinearis*), American Plaice (*Hippoglossoides platessoides*), Pollock (Pollachius virens), Snakeblenny (*Lumpenus lampretaeformis*), Longhorn Sculpin (*Myoxocephalus octodecemspinosus*), Atlantic Cod (*Gadus morhua*), Fourbeard Rockling (*Enchelyopus cimbrius*), Ocean Pout (*Zoarces americanus*), Blackbelly Rosefish (*Helicolenus dactylopterus*), Witch Flounder (*Glyptocephalus cynoglossus*), Girard’s Hagfish (*Myxine limosa*), Monkfish (*Lophius americanus*), Buckler Dory (*Zenopsis conchifer*), Haddock (*Melanogrammus aeglefinus*) and Striped Atlantic Wolffish (*Anarhichas lupus*) (Fig. 2). Among the 17 fish taxa observed, 12 are considered benthivorous (i.e. adults feeding on benthic fauna including benthic amphipods and shrimp) and five as planktivorous (i.e. adults feeding on pelagic fauna)^48,49^.

**Fig. 3:**
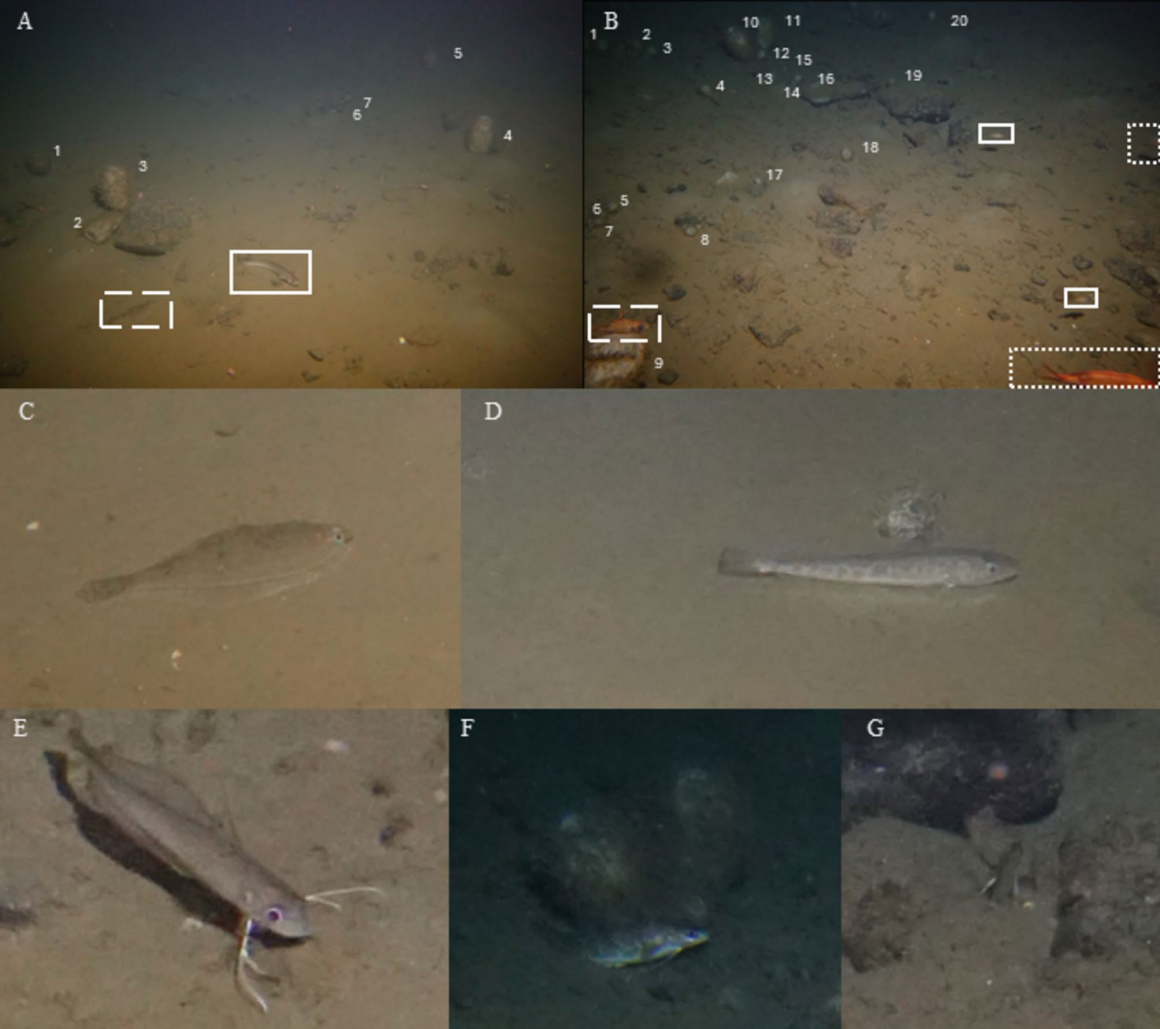
Examples of fish recorded in time-lapse images: (**A**) Image from lander M2201 showing an American Plaice (*Hippoglossoides platessoides*) (10–20 cm) (dashed box) and urophycid hake (full box) (~ 20 cm) and (**B**) Image from lander M2263 showing small (full box) (< 10 cm), medium (dashed box) (10–15 cm) and adult-sized (dotted box) (> 20cm) Redfish (*Sebastes fasciatus*). The numbers mark *Vazella pourtalesii* individuals, with (**A**) 5 large and 2 small-sized sponges at lander M2201 and (**B**) 3 large-sized and 17 small-sized sponges at lander M2263. (**C**) American Plaice (*Hippoglossoides platessoides*) (10–20 cm), (**D**) Silver Hake (Merluccius bilinearis) (10–20 cm), (**E**) Urophycid hake (< 10 cm), (**F**) Urophycid hake (~ 20 cm) using *Vazella pourtalesii* for shelter. (**G**) Urophycid hake (< 10 cm) burrowed in the sediment.

For Redfish, urophycid hake, Silver Hake and American Plaice relative size classes were noted, as observed in the images, and reproductive states were inferred. Three Redfish size classes were observed (Fig. 3B). The largest, adult-sized Redfish (> 20 cm) were observed during all three deployments, whereas medium-sized putative juveniles (10–15 cm) were only present at landers M2201 and M2263. The smallest observed juveniles, 6–10 cm, were only observed at lander M2263. The smallest juvenile Redfish (< 10 cm) and the medium and large-sized Redfish (now referred to as > 10 cm) were used in further statistical analysis. Two size classes of urophycid hakes were recorded (Fig. 3A,F,G). Medium-sized adult urophycid hakes were observed during all three deployments (~ 20 cm) (Fig. 3). Smaller-sized putative juveniles (< 10 cm) were mostly detected at lander M2263, whereas one was detected at lander M2201 (Fig. 3). More information about their observation frequency can be found in the supplementary Table 1. The observed flatfish were likely American Plaice on the basis of their morphological characteristics and evidence from nearby trawl catch surveys (Fig. 3)^50–52^. All observed Silver Hake (Fig. 3D) and American Plaice (Fig. 3A,C) were between 10 and 20 cm in length, hence they are likely juveniles^53–57^ although their reproductive state is unknown.

The acoustic receivers made 10 acoustic tag detections for which metadata on the tagged individual were available (i.e., “qualified detections”) at lander M2201, 33 at lander M2202 and 14 at lander M2263. The detections came from a total of 10 different tags, attached to four different species (Supplementary Table 2). The most common recorded species was Atlantic Bluefin Tuna (*Thunnus thynnus*), followed by a female White Shark (*Carcharodon carcharias*), Atlantic Swordfish (*Xiphias gladius*) and an Atlantic Salmon (*Salmo salar*). The tuna remained resident for the longest time of approximately two days, allowing for multiple detections.All identified species are regarded as high-speed pelagic swimmers.

Different behaviours were observed among the focal fish species. Both swimming and resting behaviours were observed in Redfish, the urophycids and Silver Hake, as determined by their presence in successive images. During 2021–22 almost all of the Redfish were observed swimming at landers M2201 (97%) and M2202 (88%), while during 2022–2023 at lander M2263, only 51% of the adult Redfish size class was swimming. Among their juveniles (< 10 cm), the majority*,* 91.4%, swam, and only 8.6% of the juveniles were resting. While the great majority of observed medium-sized urophycid hakes, 97.7%, were swimming, only 9.51% of the smaller juveniles were swimming. Some of the very small urophycid hakes sheltered or burrow themselves in the sediments (Fig. 3F,G). With only two exceptions, all Silver Hakes were classified as resting. They often remained effectively stationary, being seen in similar positions in two consecutive images, which were taken at 30 min intervals, and sometimes in more than two consecutive images. This was unusual in the other species.

### Habitat characterisation

The density of sponges in the time-lapse camera’s view and in the areas surrounding the landers, as measured with drop camera transects, differed. The fine-scale density (i.e. time-lapse camera view) of *V. pourtalesii* was highest at lander M2263 (Fig. 3B), at 1.64 individuals m^−2^, with most of the sponges being small in size. At the medium-scale, the sponge density was highest at M2201 (Supplementary Table 4). No *V. pourtalesii* sponges were visible in lander M2202 camera’s view, although they were present in the surrounding area. Cobbles and boulders were observed in the images of lander M2201 and lander M2263 (Fig. 3 A and B respectively) but not in the view of lander M2202.

Placed within 1.75 km of one another and at similar depths, the landers experienced similar oceanographic conditions (Supplementary Table 4 and Supplementary Figs. 1–2). The maximum temperature observed was 11.95 °C, recorded on 07 February 2023 at lander M2263, and the minimum 9.74 °C on 03 March 2022 at lander M2202. The highest current speed was 0.29 ms^−1^, recorded at lander M2263. All oceanographic parameter values were consistent with expectations for the Scotian Shelf’s Emerald Basin, which is flooded by an inflow of Warm Slope Water^58^. In 2022, sea-surface concentrations of Chlorophyll a (Chl a) reached their seasonal maximum in February–March (i.e. phytoplankton bloom), which coincided with the highest Monounsaturated fatty acids’ (MUFA) concentrations in the traps (Supplementary Fig. 1). The peak of the bloom occurred earlier in 2023, in December–January, and was less pronounced compared to 2022. While the Chl a and MUFA peaks were not pronounced in Feb–March 2023, the zooplankton densities and polyunsaturated fatty acids’ (PUFA) concentrations were high (Supplementary Fig. 2).

### Random forest models

Random forest (RF) model results for Redfish, urophycid hake, and Silver Hake are presented in Tables 1 and 2. Details of the fine-tuning values used to optimise the RF models are provided in the Supplementary Tables 5 and 6. The adult Redfish (> 10 cm) RF model explained most of the variability (60.83%), while the resting juvenile Redfish (< 10 cm) RF model explained the least (29.39%). The fine spatial-scale density of *V. pourtalesii* sponges was positively associated with the Redfish’s abundance among life stages. Only the model for resting juvenile Redfish (< 10 cm) featured sponge densities at other scales among its leading independent variables. Chl a, MUFA, MUFA + PUFA and (MUFA + PUFA)/SFA were all positively associated with Redfish abundance. Other indicators of food supply, such as the density of Chaetognatha and shrimp in the sediment traps were positively associated with swimming adult (10–20 + cm) and resting juvenile (< 10 cm) Redfish. Although less important than other variables, those associated with hydrodynamics also contributed to explaining the variability of Redfish abundance. Increased numbers of resting adult Redfish (> 10 cm) were associated with stronger southward currents. Small-sized swimming juvenile Redfish (< 10 cm) were associated with weaker current speeds.

**Table 1:** The percentage variability explained by each of the Random Forest models is provided in brackets.

	*Sebastes fasciatus* (> 10 cm)			*Sebastes fasciatus* (< 10 cm)		
	All (60.83%)	Swimming (55.64%)	Resting (51.22%)	All (51.46%)	Swimming (47.60%)	Resting (29.39%)
1	MUFA↑	POC/PN ↑	MUFA ↑	Fine-scale sponge density ↑	Fine-scale sponge density ↑	Fine-scale sponge density ↑
2	Month ↓	MUFA + PUFA↑	Fine-scale sponge density ↑	Salinity ↔	Salinity ↔	(MUFA + PUFA)/SFA ↑
3	Fine-scale sponge density ↑	(MUFA + PUFA)/SFA ↑	Month ↔	(MUFA + PUFA)/SFA ↑	Chl a↑	MUFA↑
4	(MUFA + PUFA)/SFA ↑	Fine-scale sponge density ↑	(MUFA + PUFA)/SFA ↑	Chl a ↑	(MUFA + PUFA)/SFA ↑	Temperature↑
5	Temperature ↑	Chaetognatha ↑	Salinity ↔	POC flux ↓	PN flux ↓	Chl a↑
6	Salinity ↔	Month ↔	Southward currents	Month ↔	Current speed ↓	200 m scale sponge density ↑
7	Shrimp↑	PUFA↑	Chl a↑	Sediment flux ↓	Temperature ↔	Zooplankton ↑
8	PN flux ↓	Chl a↑	POC/PN ↔	PN flux↓	POC flux↓	MUFA + PUFA↑
9	POC flux ↓	Copepod ↔	MUFA + PUFA ↑	MUFA + PUFA ↔	POC/PN ↔	Sponge biomass ↓
10	Zooplankton ↓	Sediment flux ↓	Zooplankton ↓	Temperature ↔	Sediment flux ↓	Shrimp↑

The Random Forest model’s top ten variables are listed in order of their highest (1) to lowest (10) percentage contribution to explaining the variability in the data, measured as the mean decrease in accuracy. The arrows next to the variables indicate indicates positive (↑), negative (↓) and non-linear, more complex ( ↔) relationships. The used abbreviations of the variables are Mono-(MUFA) and Poly (PUFA) unsaturated and saturated (SUFA) fatty acids, Particulate Organic Carbon (POC), Particulate Nitrogen (PN), Chlorophyll a (Chl a).

**Table 2:** Overview of the top ten most important environmental variables for the White Hake and Silver Hake (Merluccius bilinearis) models, in order of their % increase in mean decrease accuracy value.

	Urophycid hake (~ 20 cm) (15.28%)	Urophycid hake (< 10 cm) (55.37%)	*Merluccius bilinearis* (15.23%)
Var 1	Copepod ↓	Chl a ↑	MUFA + PUFA ↔
Var 2	PUFA ↓	PIC flux ↓	POC flux ↑
Var 3	POC/PN ↔	Current speed ↔	PN flux ↑
Var 4	Temperature ↔	Month ↔	Chl a ↔
Var 5	Zooplankton ↓	PN flux ↓	Fine-scale sponge density ↓
Var 6	Month ↔	POC flux ↓	Zooplankton ↔
Var 7	Salinity ↔	CaCO_3_ flux ↓	Chaetognatha ↔
Var 8	POC flux ↔	Salinity ↔	Sponge biomass ↑
Var 9	Sediment flux ↔	Northward current direction	PUFA ↓
Var 10	MUFA ↑	Sediment flux ↓	CaCO_3_ flux ↑

The arrows next to the variables indicate indicates positive (↑), negative (↓) and non-linear, more complex ( ↔) relationships.

The RF models of medium- (~ 20 cm) and small-sized (< 10 cm) urophycid hakes explained 15.28 to 55.37% of the variability in their abundances (Table 2). Sponge densities were not among the principal independent variables. The relationships with the variables were more complex, and not driven by positive relationships with zooplankton. Of all the RF models, the Silver Hake model explained the least variability (15.23%) (Table 2). Fine-scale sponge density was negatively associated with the fish’s abundance, but there was a positive association with broad-scale sponge-biomass density. While the relationships with the environmental variables were more complex, Silver Hake were more closely associated with patterns in zooplankton availability.

### Temporal patterns in habitat use

**Fig. 4:**
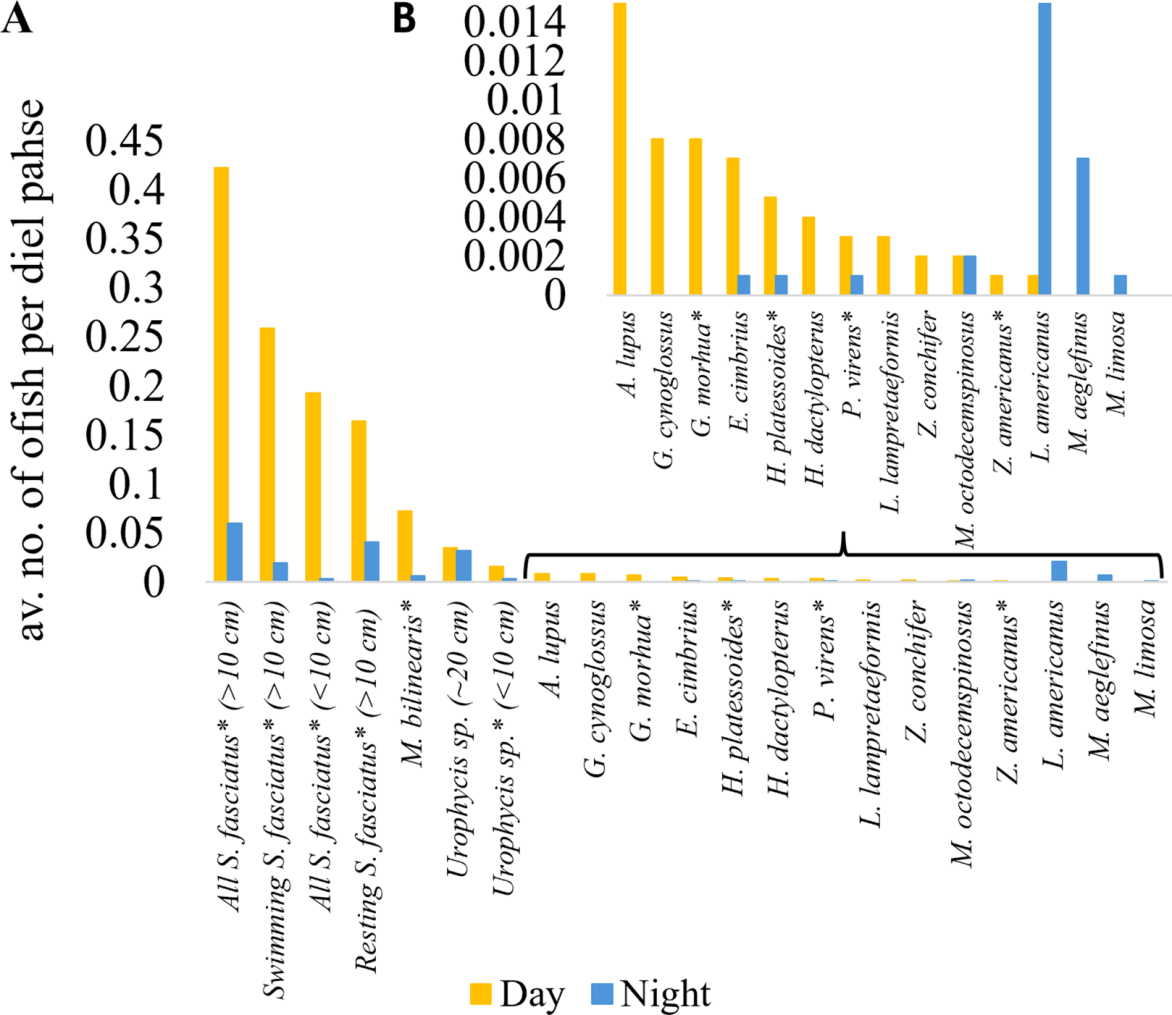
Diel patterns: The average number of fish per diel phase for each species and size class. The * next to the species names indicates where there was a significant diel difference. Fish common and Latin names: Redfish (*Sebastes fasciatus*), Silver Hake (*Merluccius bilinearis*), American Plaice (*Hippoglossoides platessoides*), Pollock (*Pollachius virens*), Snakeblenny (*Lumpenus lampretaeformis*), Longhorn Sculpin (*Myoxocephalus octodecemspinosus*), Atlantic Cod (*Gadus morhua*), Fourbeard Rockling (*Enchelyopus cimbrius*), Ocean Pout (*Zoarces americanus*), Blackbelly Rosefish (*Helicolenus dactylopterus*), Witch Flounder (*Glyptocephalus cynoglossus*), Girard’s Hagfish (*Myxine limosa*), Monkfish (*Lophius americanus*), Buckler Dory (*Zenopsis conchifer*), Haddock (*Melanogrammus aeglefinus*) and Striped Atlantic Wolffish (*Anarhichas lupus*).

**Fig. 5:**
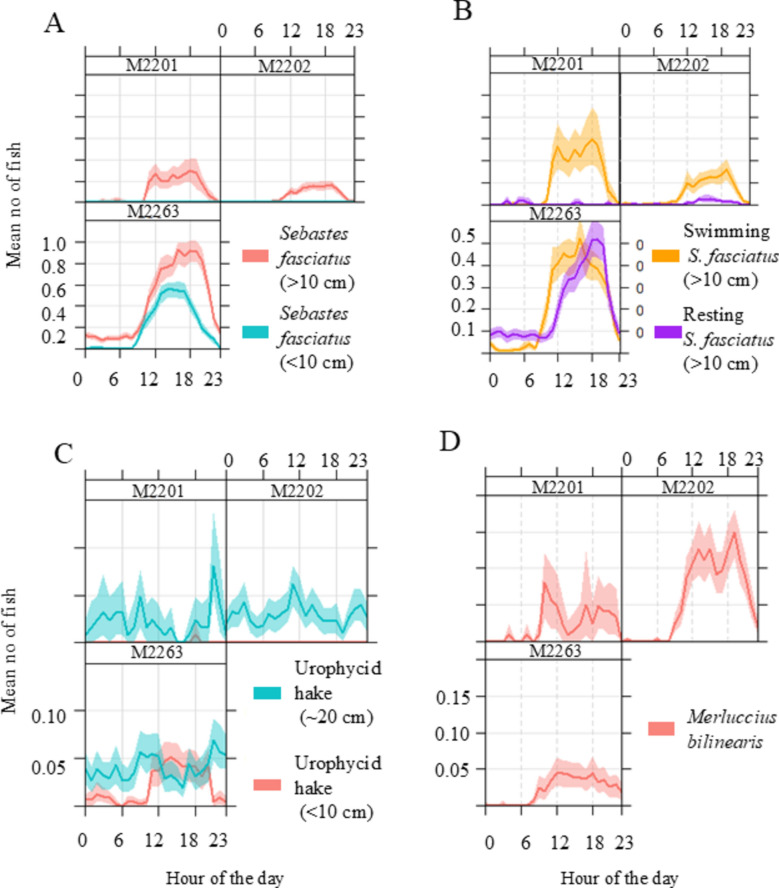
Diel patterns: Diel patterns in the mean number of observed (**A**) Redfish (*Sebastes fasciatus*), (**B**) Redfish behaviour (swimming vs resting), (**C**) Urophycid hake, and (**D**) Silver Hake (*Merluccius bilinearis*).

**Fig. 6:**
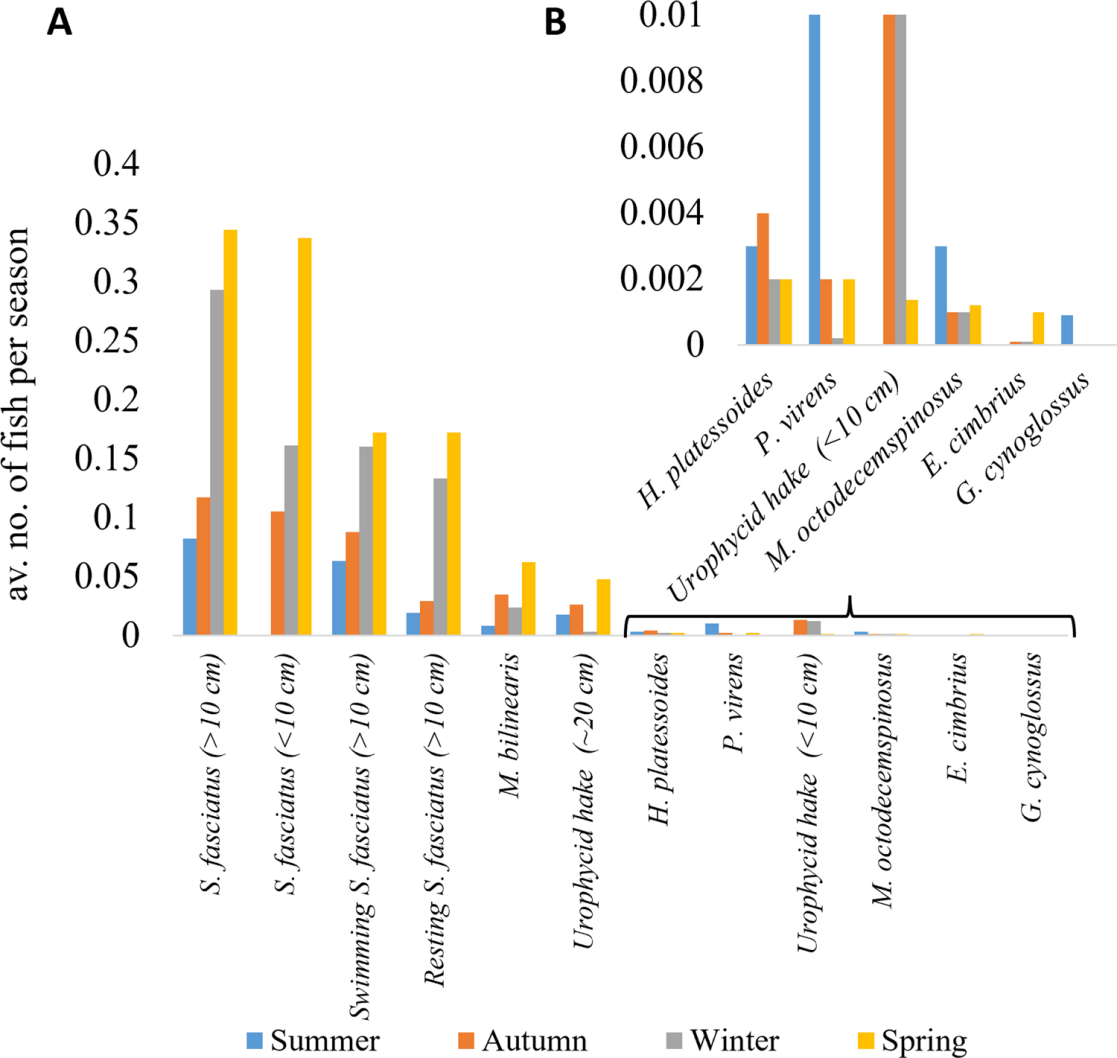
Seasonal patterns: Average number of fish per season, for each taxon and size class that had significant differences in their seasonal occurrence. Fish common and Latin names: Redfish (*Sebastes fasciatus*), Silver Hake (*Merluccius bilinearis*), American Plaice (*Hippoglossoides platessoides*), Pollock (*Pollachius virens*), Snakeblenny (*Lumpenus lampretaeformis*), Longhorn Sculpin (*Myoxocephalus octodecemspinosus*), Atlantic Cod (*Gadus morhua*), Fourbeard Rockling (*Enchelyopus cimbrius*), Ocean Pout (*Zoarces americanus*), Blackbelly Rosefish (*Helicolenus dactylopterus*), Witch Flounder (*Glyptocephalus cynoglossus*), Girard’s Hagfish (*Myxine limosa*), Monkfish (*Lophius americanus*), Buckler Dory (*Zenopsis conchifer*), Haddock (*Melanogrammus aeglefinus*) and Striped Atlantic Wolffish (*Anarhichas lupus*).

Sunrise varied between 08:36 and 11:47 and sunset between 20:33 and 23:46 (UTC). Seven of the 17 fish taxa showed significant differences in abundance between day and night, while eight others were observed in only one diel phase without reaching statistical significance due to low occurrences (Figs. 4, 5 and 6, Supplementary Table 7). All, except Monkfish, Haddock and Girard’s Hagfish, were more frequently observed on the seafloor during the day than at night. Within the Redfish’s diel cycle, a sharp increase in individuals observed on the seafloor was recorded at dawn, which decreased again at dusk (Fig. 5A,B). The dawn increase and decline at dusk were seen for all swimming Redfish, but only for resting Redfish when they were abundant enough (Fig. 5). A similar pattern was observed for Silver Hake and juvenile urophycid hake, but not for the adult urophycid hake (~ 20 cm) (Figs. 4, 5). The strength of the diel signal was greatest for the adult Redfish (d = 0.54), followed by Silver Hake (d = 0.31), juvenile urophycid hake (d = 0.13), American Plaice (d = 0.07), Atlantic Cod (d = 0.04), Ocean Pout (d = 0.04) and Pollock (d = 0.04). When comparing the Cohens’ d values per site, it is highest for Redfish by lander M2263 (d = 0.75), followed by landers M2201 (d = 0.54) and lander M2202 (d = 0.39).

Differences in abundance among the seasons were significant for eight species (Fig. 6, Supplementary Table 8). However, for the less abundant species, for which we had few observations, this pattern needs to be interpreted with caution. The observed abundance of both size classes of Redfish increased sharply from autumn to spring (Fig. 6a), while that of the urophycid hakes varied seasonally, though the pattern differed between size classes with the smaller sized individuals being more abundant in summer and autumn, and the larger sized individuals being more abundant in spring (Fig. 6a,b). Silver hake increased toward spring. American Plaice, Pollock, Longhorn Sculpin and Witch Flounder had higher numbers in summer and autumn months.

## Discussion

This study highlights how high-temporal resolution monitoring can reveal habitat usage by planktivorous and benthivorous fish on the seafloor, also known as the “Acoustic Dead Zone”^15^. A total of 17 different fish taxa used the *V. pourtalesii*-dominated sponge-ground habitat in the Sambro Bank Conservation Area, amongst which Redfish, the urophycid hakes and Silver Hake were most often observed and were recorded by all three lander deployments and across all seasons.

### Sponge grounds as nurseries

A habitat can be considered a nursery if a juvenile fish occurs at higher densities, avoids predation more successfully, or grows faster there than elsewhere^4^. This study provides the first evidence that deep and offshore sponge grounds may likely act as nurseries for four fish species, based on their high abundance. The juvenile Redfish and juvenile urophycid hakes were only observed during the second mission (2022–2023). In contrast, American Plaice juveniles were present during both missions. Because of the sexual dimorphism in the maturity of plaice it is possible that some of the juveniles recorded are actually sexually mature males^54,56,59^. Their occurrences were nearly all recorded during the study’s first mission (2021–2022), as were all Silver Hake juveniles. Fish aggregations are known to change spatially and temporally, so multiple observation station deployments are required to fully understand how the area is used over larger time and spatial scales. It is possible that a combination of both temporal and spatial factors, such as suitable oceanographic conditions, food supply, and the protection provided by benthic fauna, such as sponges, positively impacted the recruitment success of the juveniles observed in the area.

### Sponge grounds as a daytime habitat

Diel patterns for multiple species were observed. The observed changes in the on-bottom presence of Redfish are consistent with diel vertical migration behaviour, which was observed for both adults and juveniles, with the diurnal signal (the Cohens’ d value) being stronger in areas with higher sponge densities. This indicates that the association between Redfish and sponges becomes more important when sponge densities are higher. The random forest analysis also highlighted that fine-scale differences in the densities of sponges explain differences in Redfish abundances, indicating that the spatial distribution of Redfish on the seafloor may be tightly coupled to where sponges are present when they migrate to the seafloor during the daytime hours, after which they migrate upwards to feed at night^60–64^. In addition, we observed for the first time, behaviour that suggests that juvenile urophycid hake (< 10 cm), which had already metamorphosed to their benthic phase, migrate vertically during the night-time. Hakes metamorphose from pelagic larvae to benthic juveniles at about 2–4 cm length^65^. This demersal juvenile stage is not well understood. This size-class’s behaviour indicates that they use the seafloor and the pelagic realms for either shelter, food, or both. Stomach analysis studies are needed to determine this, as currently, no published studies have assessed the diet of demersal juveniles that are < 10 cm in length. After metamorphosing from larvae to juveniles, Silver Hake begins strong diurnal vertical migration and migrate upwards during the night^66–68^. This is likely linked to diel vertical plankton migrations^67^, which is supported by our results.

### Sponge grounds for feeding

Sponge grounds support a diverse range of fish and shrimp species, which can serve as benthic prey for others^13,35,45^. Redfish are long-lived (20 + years), planktivorous species, that feed mostly on pelagic prey^49,69–71^, which explains why Chl a is an important driver of their abundance. While no ‘active’ feeding was recorded, because only still images were captured, the chaetognaths and copepods collected in the sediment traps were positively associated with the occurrence of swimming Redfish, with copepods, being known as a main prey for Redfish and Chaetognaths^72^. Stomach contents have shown that increased amounts of shrimp and finfish, and fewer zooplankton are consumed when the size of Redfish increases (> 30 cm)^70,73^. This concurs with the positive relationship observed with shrimp, and the negative relationship observed with zooplankton for adult Redfish. The observed patterns extracted from the image and environmental data indicate the importance of food available on the seafloor for sustaining Redfish biomass. Silver Hake is a voracious, fast-swimming predator, that feeds on fish, crustaceans and squid, with juveniles feeding on crustacean zooplankton, chaetognaths, and shrimp. This could explain why this species was found associated with higher chaetognath and zooplankton densities^67,74^. Silver Hake was also positively associated with broader-scale sponge biomass patterns, suggesting that Silver Hake could be attracted to the sponge ground region. Sponge grounds are hotspots of nutrient cycling, which could ultimately contribute to an overall increased food supply in surface waters, which could attract these fish to the area^27,75^.

### Sponge grounds as shelters from currents

A higher number of resting adult Redfish was observed when the variability in the southwards current speeds was higher (Table 1). Other than protecting adult and juvenile fish from predators, the sponges can also act as shelters when current speeds are high^13^. It has been suggested that the protection offered by sponges gives the fish an advantage, as they can reduce the amount of energy needed to avoid displacement while resting on the seabed^13^. Almost all of the juvenile Redfish were observed swimming, which was associated with weaker currents. Few resting juvenile Redfish were recorded. This could indirectly indicate that when current speeds are high, the juvenile Redfish hide behind the sponges or engage in other cryptic behaviour not captured in the time-lapse images. Although there was no sign of a strong association between the number of urophycid hakes and sponge density, the juveniles were observed to utilise the area among the sponges when the current speeds were relatively higher (Table 1).

### Sponge grounds as shelters from predators

Sponges’ three-dimensional complexity, spicules and chemical defences can act as an anti-predatory defence mechanism (physical and chemical) against sponge-eating fish^76^ but can also protect juvenile and adult fish from predators. This study’s records of acoustically tagged fish at the study site revealed that benthic and pelagic predators such as tuna, swordfish, and white sharks occurred in the area. In addition, cannibalism by adults of many fish species, including Gadiformes (cods and hakes) and Redfish has been observed when juveniles are present in high densities^72,77,78^. Both Redfish’s size classes had positive associations with sponge density at fine spatial scales. In addition to this, urophycid juveniles were also observed burrowing in the sediments near sponges and boulders (Fig. 3). Sand-hiding behaviour has been observed for White Hake in shallow environments, and observed burrowing behaviour in sandy, eelgrass, and rocky habitats^79^. It has been suggested that micro-habitats, such as burrows act as refuges from predators or may be an ambush station when hunting for food^80^, with the sponges providing additional protection. While no positive association was found between sponge density at any scale and Silver Hake abundance, this does not rule out that, on occasion, the juveniles may use the sponges for protection from predators. Observations of predator–prey interactions are rare and difficult to assess from images alone. However, the positive associations found in this study indicate that *Vazella pourtalesii,* which has large silica spicules that extend beyond their epidermis^81^, could act as a place to shelter from predators for fish, which was confirmed by Hawkes et al. 2019.

### Benthic-landers as “early-warning” tools for sustainable fishery management

The trends in the differences of fish abundances between the different years observed in this study’s images were aligned with the subsequent fish stock assessments made in the summers of 2023 and 2024^50,51,82^. For example, as predicted from the images of Redfish juveniles from Autumn 2022 to Spring 2023, the DFO summer 2023 fishery survey recorded higher captures of 15–20 cm size class Redfish compared to the previous year, when these were predominantly 10–15 cm. Additionally, the summer 2023 fishery survey yielded specimens of very small Redfish, measuring 5–10 cm, which would have been consistent with the specimens seen earlier in the year (spring 2023) in the images. To effectively inform fishery management and predict how species use vulnerable benthic habitats as a nursery, shelter and feeding ground, and respond to climate change and human pressures, interdisciplinary thinking and using cost-effective non-destructive monitoring approaches, such as benthic lander observation stations, are needed^83,84^. Other novel approaches include environmental DNA^85–87^ and passive acoustic monitoring^88,89^. When real-time monitoring and the automation of data analysis are integrated, benthic observation stations could act as a cost effective and non-destructive “early-warning” monitoring tool to indicate changes in fish populations, which could, in turn, inform fishery management^90–92^. Using an ecosystem-based monitoring approach is fundamental to understanding how changes in the environment impact our natural resources and is key for developing and implementing more robust evidence-based policy and management decisions^93^.

## Methods

### Benthic landers

#### Lander deployments

Each benthic lander comprised a triangular aluminium frame, supporting the instrument packages, with steel weights attached via an acoustic release, plus plastic-cased glass floats. A strobe light, radio beacon and satellite beacon were included to aid recovery^47,94,95^.

Three landers, M2201, M2202 and M2263 were deployed in sponge grounds within the Sambro Bank Conservation Area from September 2021 until May 2022 (Fig. 1), although one contained non-comparable environmental data and therefore, the data from only two landers were analysed here, i.e. M2201 and M2202. Thereafter, one lander was redeployed from October 2022 to July 2023, i.e. lander M2263 (Fig. 1)^47,94,95^.

#### Lander instruments

Details of the instruments on each lander are provided in the Supplementary Tables 9 and 10. They included a time-lapse camera with flash lighting, a Technicap PPS 4/3 Sediment Trap (net volume: 12 × 250 ml or 6 L), a Seabird MicroCAT 37 Conductivity, Temperature, Depth (CTD) sensor, an upward-looking Sentinel V Acoustic Doppler Current Profiler (ADCP) V100 (307 kHz), and an Ocean Tracking Network (OTN) receiver to detect acoustically tagged fishes^96^. Time-lapse images were collected every 30 min, the CTDs were scheduled to record every 15 min and the ADCPs recorded an ensemble of 26 pings over a 13-s interval every 20 min along a 2 m bin size. The sediment traps collected sinking particles over periods of 14 to 28 days, bottles 1–3 collected sediment for two weeks each, bottles 4–9 for four weeks and bottles 10–12 for two weeks. The landers also carried passive acoustic monitoring devices, but this study did not consider the resulting data^47,94,95^.

### Data processing

#### Time-lapse images

The seabed area falling within the field of view of each time-lapse camera was ~ 12.20 m^2^ calculated using Inventor software (Supplementary Fig. 3 and 4). This estimation of the lander’s view area was also used to estimate the size classes of the fish and sponges (Supplementary Fig. 3). The small (< 80 mm) and large (> 80 mm) *V. pourtalesii* visible within that area were counted and the fine-scale densities (i.e. at the time-lapse image camera’s view) of the sponges were determined.

All the fish that appeared in the images were identified to the lowest possible taxonomic level and annotated in BIIGLE 2.0^97^ a web tool for image annotation^98,99^. For the few abundant species, counts were made separately for various size classes. Individuals less than 20 cm are generally considered juveniles for several fish species. Those seen in images were not measured but categorised into different size classes, using Supplementary Fig. 3 and measurements conducted during the regional fisheries surveys as guidance (DFO 2022, 2023, 2024). For example, very small redfish were less red, corresponding to the 6–10 cm size class in fishery captures, while red ones were of the 14–20 cm class, and large, deep-bodied individuals would belong to the adult class of 22–30 cm, which were the Redfish groupings sampled in summer 2023, as can be seen in Fig. 9e in the Fisheries and Oceans Canada (DFO) 2024 survey report^51^. Since it was not always possible to distinguish between the medium and large size classes, they were grouped for analysis and hereafter referred to as “adult Redfish (> 10 cm)” (Fig. 3B). The small juvenile Redfish are referred to as the < 10 cm size class. The numbers of each taxon or size class were reduced to daily mean values to minimise temporal pseudoreplication arising from the same individuals being present in multiple images^100^. Observations of fish behaviour were noted and were categorised into swimming (including hovering and drifting) or resting^97^. Swimming was when an individual in still images exhibited some motion blur, while resting was presumed when it was on or near the bottom, and certain when seen in the same position over several images. Pandalid shrimps *(Dichelopandalus leptocerus* and *Pandalus montagui*), potential prey items for the fish, were also counted in the time-lapse images and included in the analysis. DFO Science Advisory Reports, reporting fishery data collected from 2021 to 2024, in the area surrounding the Sambro Bank Conservation Area, were consulted to support species identification and size determination.

#### OTN records

Coded acoustic signals identifying each tagged animal that passed within the approximately 500 m detection range of the OTN receiver were logged. The data were subsequently uploaded to a central database, resulting in current and reliable global records^101^. The tracker code, date and time of the tagged fish approaching the receivers were recorded. The data from this study can be found in the OTN project SPONGE—OTN Sponge Ground Landers^94^.

#### CTD and ADCP data

The CTD data were processed with Seabird Data Processing software 1.59, followed by analysis in MATLAB with the Gibbs SeaWater (GSW) Oceanographic Toolbox of TEOS-10 (https://www.teos-10.org/software.html). The raw ADCP data were processed using RDI’s Velocity Software 1.7.21 to generate values in MATLAB. Those outputs were ensemble-averaged, generating hourly profiles, with a vertical range of 94 m from the transducer face. These datasets were carefully examined for outliers, and low-quality data were excluded from further RF analysis.

#### Sediment trap data

The collected material was analysed for particulate organic matter and lipid biomarkers. The concentrations and fluxes of sediment, particulate organic carbon (POC), particulate organic nitrogen (PN), calcium carbonate (CaCO_3_), and the lipid components, namely monounsaturated fatty acids (MUFA), polyunsaturated fatty acids (PUFA) and saturated fatty acids (SFA) were derived from the sediment-trap samples. The ratios of several variables (POC/PN, MUFA/PUFA, (MUFA + PUFA)/SFA) were calculated. These variables give an indication of the abundance and quality of food in the area. More details on how the samples were processed can be found in de Froe et al.^102^. Zooplankton found in the sediment-trap samples were sorted into broad taxonomic groups and counted. The number of chaetognaths, copepods, annelids and total zooplankton were divided by 14 or 28, depending on the sample (see lander instruments), to establish an estimate of daily average abundances.

### Other data sources

#### Satellite imaging

The monthly average chlorophyll a concentrations (Chl a) (mg m^−3^) recorded with the Aqua-MODIS satellite were downloaded from the NASA ocean colour portal, with a resolution of 4 × 4 km^103^. The Extract Values to Points tool in ArcPro v2.9 was used to extract the average monthly Chl a concentrations per lander site.

#### Drop-camera surveys

In addition to the fine-scale (i.e. from the time-lapse images) and the broad-scale sponge densities (from the modelled trawl data, see below), medium-scale densities (individuals m^−2^) of both small and large *V. pourtalesii* were determined at two spatial scales from drop-camera surveys. Surveys were conducted along crossed transects surrounding the benthic lander deployment sites using a drop camera with an ~ 0.86 m^2^ field of view^47,94,95^. The sponge densities were averaged inside a 0–100 m and a 0–200 m radius around each lander site. The Buffer tool in ArcGIS Pro v2.9.5 was used to outline the radii. Although the sizes of the chosen radii are arbitrary, they were chosen to determine if scale had an influence.

#### Trawl surveys

Broad-scale densities of sponges, which were calculated on the basis of modelled results from trawl survey catch data were quantified by extracting the sponge biomass (1 × 1 km resolution)^104^ and the likelihood of *V. pourtalesii* presence^105^ within a one-kilometre radius around each lander site, using the Buffer and Extract Values to Points tools in ArcPro v2.9.5.

### Statistical analyses

For Redfish (in two size classes and behavioural groupings), urophycid hakes (two size classes) and Silver Hake, which are the three most frequently observed taxa, daily mean fish abundances were assessed against multiple independent variables, including sponge densities at fine, medium and broad spatial scales, various measures derived from sediment traps, ocean current velocity and direction, temperature, salinity, surface Chl a and month, with the latter capturing the seasonal cycles in fish migrations and behaviours. To understand what drives each fish taxon and size class’ behaviour, a model was produced for each category, resulting in nine Random Forest (RF) regressions, using the ‘RandomForest’^106^ and ‘caret’ packages^107^ in R v4.4.0. Model performance was optimised by adjusting the numbers of trees (ntree), iterations (mtry) and terminal nodes (maxnodes). Model accuracy was assessed by training models on 70% of the data, applying those models to the remaining 30% and calculating the Root-Mean-Squared-Error^108^. The percentage variance explained by the models is also reported. Understanding these relationships was aided by examining partial response curves of each predictor variable.

To determine whether diel (categorised as daylight vs. night) or seasonal (categorised as autumn vs. winter vs. spring vs. summer) patterns occurred, records were classified by season and diel phase. For the latter, sunrise and sunset times were calculated for each day and location using the ‘bioRad’ package in R^109^. Seasons were categorised as “summer”, starting 21 June, “autumn”, starting 22 September, “winter” starting 21 December and “spring” starting 20 March. To determine if there are significant differences in the fish abundances between diel phase and season, Kruskal Wallis tests were conducted due to the absence of normality in the distribution of the data. Significant diel and seasonal patterns were visualised using the *timeVariation* function from the ‘openair’ package in R^110^. To quantify the strength of diel and seasonal differences in the fish abundances, the Cohen’s d (d) metric (“lsr” package) was calculated^111^.

## Supplementary Information

Supplementary Information.

## Data Availability

The data that support the findings of this study have been deposited in the Mendeley database (10.17632/dfbmyr36kp.1), which is an open-access platform from which the data can be downloaded. Laurence H. De Clippele (email: laurence.declippele@glasgow.ac.uk) is the point of contact.
