# Supplementary material for: Fish use of deep-sea sponge habitats evidenced by long-term high-resolution monitoring
Source: Sci Rep. 2025 May 21;15:17656. doi: 10.1038/s41598-025-01822-5 (PMC12095674; doi:10.1038/s41598-025-01822-5)

**Supplementary Materials**

**Fish use of deep-sea sponge habitats evidenced by long-term high-resolution monitoring**

*Laurence H. De Clippele^1*^, Claude Nozères^2^, Jinshan Xu^2^, Barry MacDonald^2^, Camille Lirette^2^, Kirk Phelan^2^, Calisa Staniforth^2^, Fred Whoriskey^3^, George A. Wolff^4^, Sabena Blackbird^4^, Christian Mohn^5^, Ellen Kenchington^2^*

*^1* School of Biodiversity, One Health and Veterinary Medicine, University of Glasgow, G12 8QQ, UK, laurence.declippele@glasgow.ac.uk^*

*^2 Department of Fisheries and Oceans, Bedford Institute of Oceanography, Dartmouth, Nova Scotia B2Y4A2 Canada^*

*^3 Ocean Tracking Network, Dalhousie University, Halifax, NS, Canada^*

*^4 School of Environmental Sciences, University of Liverpool, Liverpool, L69 3GP^*

*^5 Department of Ecoscience, Aarhus University, Roskilde, Denmark^*

Table 1 of the total number of occurrences (note that some may be the same individual over consecutive images) and average (av.) number of occurrences ± standard deviation of adult and juvenile (Juv) fish species seen in time-lapse images, per lander site (LS).

|  | No. of occurrences | | | | Av. (± stdev) no. occurrences per image | | | |
| --- | --- | --- | --- | --- | --- | --- | --- | --- |
| SPECIES | **LS1** M2201 | **LS2** M2202 | **LS3** M2179 | **LS1** M2263 | **LS1**  M2201 | **LS2**  M2202 | **LS3**  M2179 | **LS1** M2263 |
| American Plaice (10-20 cm)  (*Hippoglossoides platessoides*) | 11 | 53 | 1 | 2 | 0.004 ± 0.061 | 0.013  ± 0.114 | 0.009x10^-2^  ± 0.01 | 0.002 x10^-1^  ± 0.013 |
| Atlantic Cod  (*Gadus morhua*) | 4 | 1 | 0 | 3 | 0.001  ± 0.037 | 0.001 x10^-1^  ± 0.009 | 0 | 0.003 x10^-1^  ± 0.016 |
| Striped Atlantic Wolffish  (*Anarhichas lupus*) | 0 | 1 | 0 | 0 | 0 | 0.001 x10^-1^  ± 0.009 | 0 | 0 |
| Blackbelly Rosefish  *(Helicolenus dactylopterus*) | 0 | 0 | 0 | 4 | 0 | 0 | 0 | 0.003 x10^-1^  ± 0.018 |
| Buckler Dory  (*Zenopsis conchifer*) | 0 | 0 | 0 | 2 | 0 | 0 | 0 | 0.001 x10^-1^  ± 0.013 |
| Fourbeard Rockling  (*Enchelyopus cimbrius*) | 0 | 6 | 23 | 2 | 0 | 0.001  ± 0.023 | 0.002  ± 0.044 | 0.002 x10^-1^  ± 0.013 |
| Girard’s Hagfish  (*Myxine limosa*) | 0 | 0 | 22 | 2 | 0 | 0 | 0.002  ± 0.043 | 0.002 x10^-1^  ± 0.013 |
| Haddock  (*Melanogrammus aeglefinus*) | 0 | 0 | 0 | 1 | 0 | 0 | 0 | 0.001 x10^-1^  ± 0.009 |
| Longhorn Sculpin  (*Myoxocephalus octodecemspinosus*) | 4 | 1 | 0 | 6 | 0.001  ± 0.037 | 0.001 x10^-1^  ± 0.009 | 0 | 0.001  ± 0.023 |
| Monkfish  (*Lophius americanus*) | 0 | 1 | 5 | 1 | 0 | 0.001 x10^-1^  ± 0.009 | 0.004x10^-1^  ± 0.021 | 0.001 x10^-1^  ± 0.009 |
| Ocean Pout  (*Zoarces americanus*) | 0 | 1 | 0 | 8 | 0 | 0.001 x10^-1^  ± 0.009 | 0 | 0.001  ± 0.026 |
| Pollock  (*Pollachius virens*) | 11 | 16 | 1 | 10 | 0.004  ± 0.066 | 0.001  ± 0.082 | 0.001 x10^-2^  ± 0.009 | 0.001  ± 0.066 |
| Redfish (>10 cm)  (*Sebastes fasciatus*) | 316 | 681 | 181 | 5,155 | 0.106  ± 0.400 | 0.059 ± 0.299 | 0.016± 0.160 | 0.436  ± 0.942 |
| Redfish (<10 cm)  (*Sebastes fasciatus*) | 0 | 0 | 0 | 2,404 | 0 | 0 | 0 | 0.203 ± 0.610 |
| Silver Hake (10-20 cm) (*Merluccius bilinearis*) | 64 | 654 | 204 | 256 | 0.022  ± 0.158 | 0.056  ± 0.277 | 0.018  ± 0.141 | 0.022  ± 0.150 |
| Snakeblenny  (*Lumpenus lampretaeformis*) | 0 | 3 | 0 | 0 | 0 | 0.003  ± 0.016 | 0 | 0 |
| Urophycid hake (10-20 cm)  (*Urophycis* sp.) | 64 | 348 | 268 | 22 | 0.022  ± 0.147 | 0.030  ± 0.177 | 0.023  ± 0.151 | 0.200  ± 0.040 |
| Urophycid hake (<10 cm)  (*Urophycis* sp.) | 1 | 0 | 0 | 249 | 0.003x10^-1^  ± 0.018 | 0 | 0 | 0.018  ± 0.136 |
| Witch Flounder  (*Glyptocephalus cynoglossus*) | 1 | 0 | 0 | 0 | 0.0003x10^-1^  ± 0.018 | 0 | 0 | 0 |

Table 2 OTN species records, tracker project code, location, number of sensors (= no. of indiv.), dates and time range at which records were recorded.

| **Species** | **TrackerCode** | **Site** | **No indiv. tags** | **Dates** | **Time** |
| --- | --- | --- | --- | --- | --- |
| **Atlantic Bluefin Tuna** | [TAG](https://eur03.safelinks.protection.outlook.com/?url=https%3A%2F%2Fmembers.oceantrack.org%2Fproject%3Fccode%3DTAG&data=05%7C02%7CLaurence.DeClippele%40glasgow.ac.uk%7C5a90782ad2e04a159b5d08dc3863dcc0%7C6e725c29763a4f5081f22e254f0133c8%7C1%7C0%7C638447248770129691%7CUnknown%7CTWFpbGZsb3d8eyJWIjoiMC4wLjAwMDAiLCJQIjoiV2luMzIiLCJBTiI6Ik1haWwiLCJXVCI6Mn0%3D%7C0%7C%7C%7C&sdata=o7BJjA2Zl7JVcLnPkzyBWNq9DYZ7BFGWTGHvRZ4gLJI%3D&reserved=0) | LS1 M2201 | 1 | 10/10/2021  12/10/2021  04/11/2021 | 13:33-13:34  14:58-15:02  14:56-14:59 |
|  |  | LS2 M2202 | 2 | 10/10/2021  12/10/2021  15/06/2022 | 13:41-17:12  15:22-16:02  09:39 |
|  |  | LS3 M2179 | 0 | 0 | 0 |
|  |  | LS1 M2263 | 1 | 10/10/2023 | 10:54-11:10 |
| **White Shark** | [ACT.MSRPWS](https://members.oceantrack.org/project?ccode=ACT.MSRPWS) | LS1 M2201 | 2 | 09/12/2021 | 21:31-21:34 |
|  |  | LS2 M2202 | 2 | 09/12/2021 | 21:17-21:19 |
|  |  | LS1 M2263 | 0 | 0 | 0 |
| **Atlantic Swordfish** | [DFOBFT](https://members.oceantrack.org/project?ccode=DFOBFT) | LS1 M2201 | 0 | 0 | 0 |
|  |  | LS2 M2202 | 1 | 13/11/2021 | 20:53-20:55 |
|  |  | LS3 M2179 | 1 | 06/11/2021  15/11/2021 | 08:06-09:12  16:47-17:09 |
|  |  | LS1 M2263 | 0 | 0 | 0 |
| **Atlantic Salmon** | [ESRF](https://members.oceantrack.org/project?ccode=ESRF) | LS1 M2201 | 0 | 0 | 0 |
|  |  | LS2 M2202 | 0 | 0 | 0 |
|  |  | LS3 M2179 | 0 | 0 | 0 |
|  |  | LS1 M2263 | 1 | 12/05/2023 | 09:37-09:49 |
| **Blue Shark** | [NSBS](https://members.oceantrack.org/project?ccode=NSBS) | LS1 M2201 | 0 | 0 | 0 |
|  |  | LS2 M2202 | 0 | 0 | 0 |
|  |  | LS3 M2179 | 1 | 05/11/2021 | 21:18-22:12 |
|  |  | LS1 M2263 | 0 | 0 | 0 |

Table 3 The total number of occurrences for each behaviour. Overview of the total number of occurrences and average (av.) number of occurrences ± standard deviation of fish behaviour and size classes seen in time-lapse images, per lander site (LS).

|  | Total no. occurrences | | | | av. (± stdev) no. occurrences per image | | | |
| --- | --- | --- | --- | --- | --- | --- | --- | --- |
|  | **LS1**  M2201 | **LS2**  M2202 | **LS3**  M2179 | **LS1**  M2263 | **LS1**  M2201 | **LS2**  M2202 | **LS3**  M2179 | **LS1**  M2263 |
| **Redfish adult resting (>10 cm)** | 10 | 81 | 90 | 2,490 | 0.003  ± 0.058 | 0.007 ± 0.093 | 0.008 ± 0.113 | 0.184 ± 0.568 |
| **Redfish adult swimming (>10 cm)** | 306 | 600 | 91 | 2,550 | 0.103 ± 0.397 | 0.052 ± 0.280 | 0.008 ± 0.110 | 0.189 ± 0.619 |
| **Redfish**  **Swimming (<10 cm)** | 0 | 0 | 0 | 2,195 | 0 | 0 | 0 | 0.186 ± 0.592 |
| **Redfish (<10 cm)** | 0 | 0 | 0 | 208 | 0 | 0 | 0 | 0.018 ± 0.136 |
| **Resting urophycid hake (~20 cm)** | 0 | 0 | 0 | 20 | 0 | 0 | 0 | 0.002 ± 0.0410 |
| **Swimming urophycid hake adult swimming (~20 cm)** | 64 | 348 | 268 | 448 | 0.022  ± 0.147 | 0.030 ± 0.177 | 0.023 ± 0.151 | 0.0379 ± 0.196 |
| **Resting urophycid hake (<10 cm)** | 1 | 0 | 0 | 219 | 0.003x10^-1^  ± 0.018 | 0 | 0 | 0.019 ± 0.136 |
| **Swimming urophycid swimming (<10 cm)** | 0 | 0 | 0 | 22 | 0 | 0 | 0 | 0.020 ±.045 |

*Table 4* Overview of environmental variables per benthic lander deployment: Total/small (< 80 mm)/large (> 80 mm) sponge densities of *V. pourtalesii* at fine, medium and broad (presence/absence and biomass); average ± standard deviation of oceanographic datasets such as temperature, salinity, current direction and speed. Indicators of food supply are represented by Chlorophyll a, mono (MUFA) and poly (PUFA) unsaturated fatty acids, saturated fatty acids (SFA), particulate organic (POC), nitrogen (PN), calcium carbonate (CaCO3) and sediment. Zooplankton records included, average abundances of annelids, chaetognaths, copepods and shrimp. The latter included pandalid abundance records recorded in the images.

| **Environmental data** | **M2201** | **M2202** | **M2263** |
| --- | --- | --- | --- |
| Fine-scale sponge density  *Small/Large sized sponges* | 0.57 | 0 | 1.64 |
|  | *0.25/ 0.33* | *0* | *1.39/0.25* |
| 100 m-scale sponge density  *Small/Large sized sponges* | 1.74 | 0.10 | 0.73 |
|  | *1.55/0.19* | *0/0.10* | *0.44/0.29* |
| 200 m -scale sponge density  *Small/Large sized sponges* | 2.11 | 0.27 | 0.86 |
|  | *1.45/0.65* | *0.20/0.07* | *0.68/0.18* |
| Sponge’s likelihood of presence | 0.94 | 0.97 | 0.94 |
| Sponge biomass (kg km^-2^) | 10.79 | 13.53 | 10.79 |
| Bottom temperature (C) | 11.21 ± 0.13 | 10.80 ± 0.56 | 11.12± 0.24 |
| Salinity (psu) | 35.04 ± 0.15 | 35.04 ± 0.14 | 34.99 ± 0.10 |
| Current direction (°) | 173.26 ± 58.35 | 178.8 ± 43.82 | 199.77± 64.20 |
| Current Speed (m s^-1^) | 0.073 ± 0.019 | 0.078 ± 0.023 | 0.105 ± 0.028 |
| Chl a (mg m^−3^) | 0.708 ± 0.145 | 1.243 ±0.593 | 1.055 ± 0.263 |
| MUFA (gr) | 20.115 ± 0.955 | 10.544 ± 6.423 | 22.751 ± 7.218 |
| PUFA (gr) | 5.067 ± 1.381 | 2.6 ± 3.517 | 6.605 ± 3.702 |
| SFA (gr) | 18.086 ± 8.033 | 13.353 ±13.08 | 10.246 ± 5.440 |
| MUFA+ PUFA (gr) | 21.250 ± 9.846 | 5.976 ± 8.520 | 21.116 ± 12.759 |
| (MUFA+ PUFA)/SFA (gr) | 1.155 ± 0.372 | 0.355 ± 0.255 | 1.992 ± 0.764 |
| POC Flux mmol | 5.453 ± 2.016 | 8.466 ± 3.964 | 6.522 ± 4.107 |
| PN Flux mmol | 0.635 ± 0.206 | 0.957 ± 0.441 | 0.789 ± 0.521 |
| POC/PN | 8.368 ± 0.657 | 8.864 ± 0.819 | 8.351 ± 0.699 |
| CaCO_3_ Flux mmol | 7.375 ± 2.159 | 12.37 ± 7.343 | 8.978 ± 6.814 |
| Sediment Flux mmol | 1.592 ± 0.679 | 2.924 ± 1.646 | 1.905 ± 1.350 |
| Annelids (indiv. day^-1^) | 0.054 ± 0.59 | 0.017 ± 0.039 | 0.018 ± 0.028 |
| Chaetognath (indiv. day^-1^) | 0.018 ± 0.031 | 0.008 ± 0.023 | 0.080 ± 0.114 |
| Copepod (indiv. day^-1^) | 1.047 ± 0.639 | 0.328 ± 0.412 | 0.652 ± 0.283 |
| Zooplankton (indiv. day^-1^) | 1.208 ± 0.544 | 0.425 ± 0.470 | 0.826 ± 0.262 |
| Shrimp (indiv. day^-1^) | 0.046 ± 0.060 | 0.036 ± 0.078 | 0.084 ± 0.168 |

*Figure 1* Temporal variability in Chlorophyll a and zooplankton: Chlorophyll a (Chl a) and zooplankton (Zo) density at the different deployment locations (M2201, M2202, M2263) over time. The numbers on the x-axis represent the sediment trap samples which were collected over periods of 14 to 28 days.


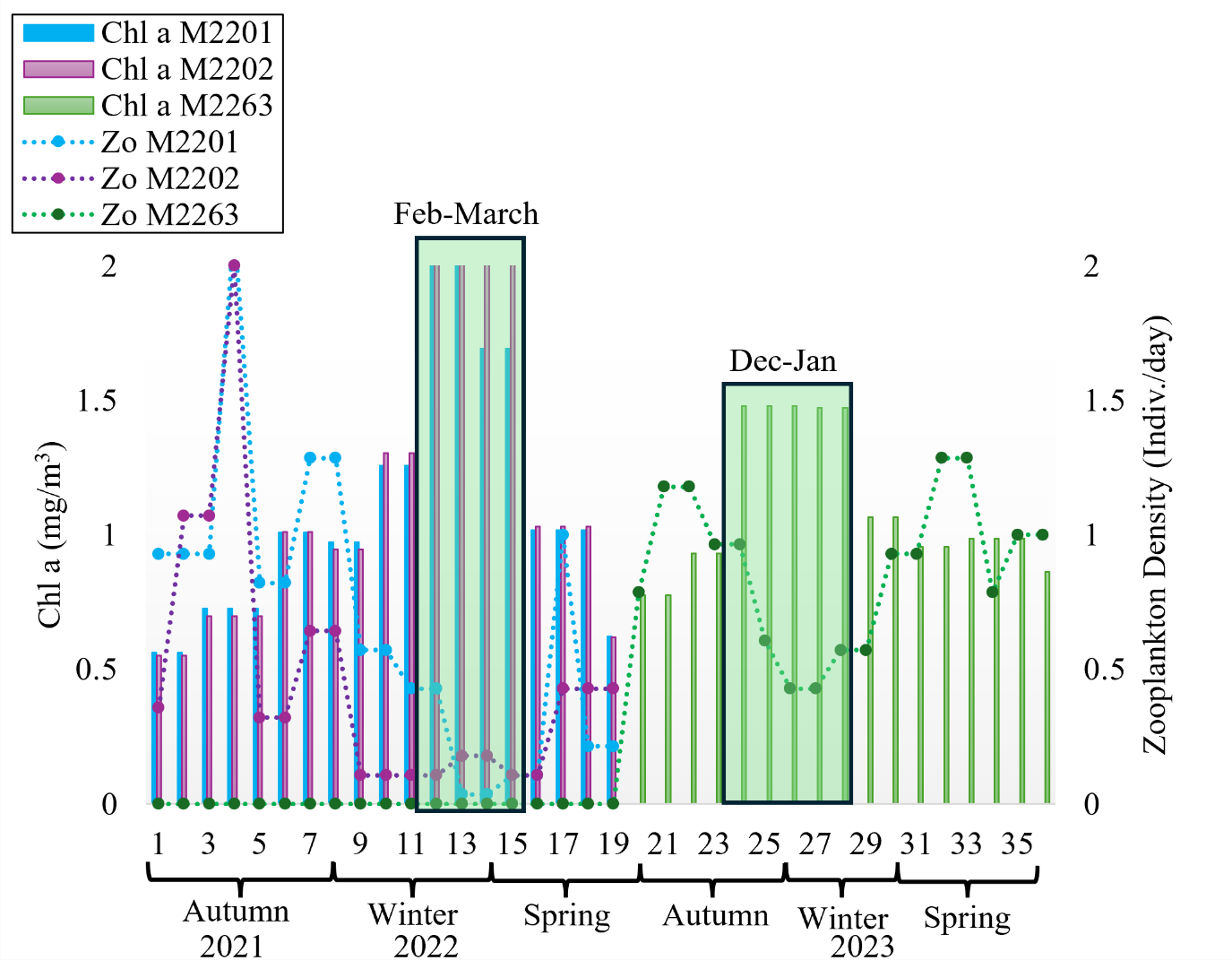


*Figure 2* Temporal variability in MUFAs and PUFAs: Mono- (MUFA) and poly (PUFA) unsaturated fatty acid concentrations at the different deployment locations (M2201, M2202, M2263) over time. The numbers on the x-axis represent the sediment trap samples which were collected over periods of 14 to 28 days.


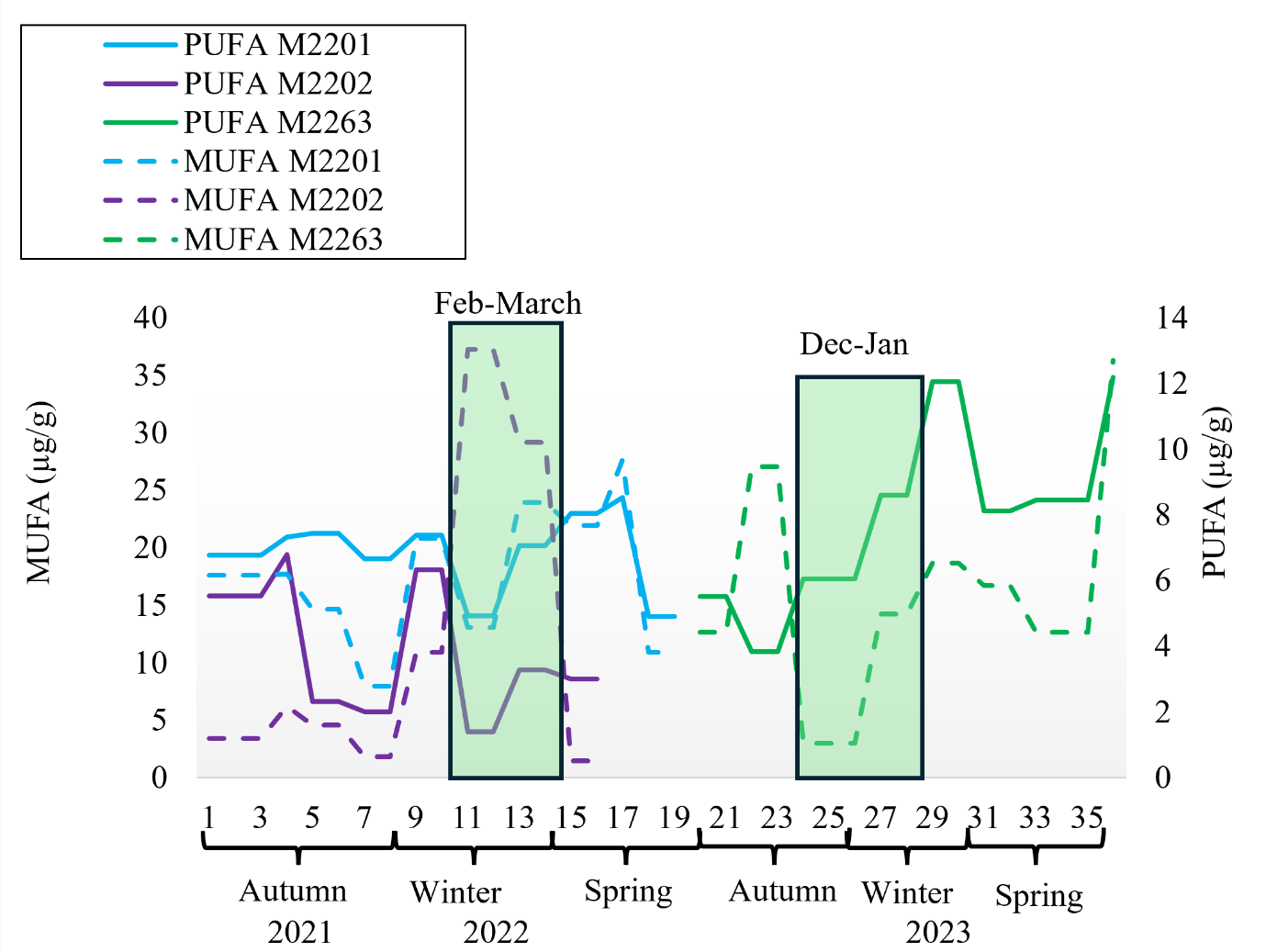


Table 5 Random forest model parameters for the Redfish models, the % variability (var.) explained by the model, the root-mean-square-deviation (RMSE) for the model trained on the training data (80% of the data) and the RMSE when testing the model on the test data.

|  | **Redfish Adults** | **Redfish (10-20+ cm)**  **Swim** | **Redfish**  **(10-20+ cm)Rest** | **Redfish.**  **(<10 cm)** | **Redfish**  **(<10 cm) Swim** | **Redfish (<10 cm)Rest** |
| --- | --- | --- | --- | --- | --- | --- |
| **mtry** | 10 | 2 | 9 | 5 | 3 | 2 |
| **maxnodes** | 35 | 19 | 22 | 30 | 39 | 23 |
| **ntree** | 100 | 100 | 300 | 250 | 600 | 500 |
| **var. explained (%)** | 60.83 | 55.64 | 51.22 | 51.46 | 47.60 | 29.39 |
| **RMSE default model** | 0.163 | 0.095 | 0.114 | 0.081 | 0.074 | 0.021 |
| **RMSE validation** | 0.241 | 0.192 | 0.112 | 0.229 | 0.236 | 0.287 |

Table 6 Random forest model parameters for the phycid hake and Silver Hake models, the % variability (var.) explained by the model, the root-mean-square-deviation (RMSE) for the model trained on the training data (80% of the data) and the RMSE when testing the model on the test data.

|  | **Urophycid hake (~20cm)** | **Urophycid hake (<10cm)** | **Silver Hake** |
| --- | --- | --- | --- |
| mtry | 1 | 10 | 2 |
| maxnodes | 18 | 50 | 7 |
| ntree | 2000 | 100 | 100 |
| var. explained (%) | 15.28 | 55.37 | 15.23 |
| RMSE default model | 0.032 | 0.026 | 0.075 |
| RMSE validation | 0.275 | 0.289 | 0.285 |

Table 7 Diurnal patterns, results from Kruskall wallis test, the average +- stdev of the abundances and the Cohen’s d values

|  | **Diel** | **Day** | **Night** | **Cohen’s d** |
| --- | --- | --- | --- | --- |
| American Plaice  (*Hippoglossoides platessoides*) | **<0.005** | 0.004  ± 0.065 | 0.001  ± 0.030 | 0.07 |
| Atlantic Cod  (*Gadus morhua*) | **<0.005** | 0.007x10^-1^  ± 0.026 | 0 | 0.04 |
| Atlantic Wolffish  (*Anarhichas lupus*) | 0.282 | 0.008 x10^-20^ ± 0.009 | 0 | 0.01 |
| Blackbelly Rosefish  (*Helicolenus dactylopterus*) | 0.032 | 0.003 ± 0.018 | 0 | 0.03 |
| Buckler Dory  (*Zenopsis conchifer*) | 0.128 | 0.002 x10^-1^ ± 0.013 | 0 | 0.02 |
| Fourbeard Rockling  (*Enchelyopus cimbrius*) | 0.105 | 0.005 ± 0.022 | 0.001 x10^-1^ ± 0.012 | 0.02 |
| Girard’s Hagfish  (*Myxine limosa)* | 0.188 | 0 | 0.001 x10^-1^ ± 0.012 | 0.02 |
| Haddock  (*Melanogrammus aeglefinus*) | 0.352 | 0 | 0.007 x10^-1^ ± 0.008 | 0.01 |
| Longhorn Sculpin  (*Myoxocephalus octodecemspinosus*) | 0.08 | 0.001  ± 0.025 | 0.002 x10^-1^ ± 0.015 | 0.02 |
| Monkfish  (*Lophius americanus*) | 0.18 | 0 | 0.021 x10^-2^  ± 0.012 | 0.02 |
| Ocean Pout  (*Zoarces americanus*) | **0.001** | 0.001  ± 0.027 | 0 | 0.04 |
| Pollock  (*Pollachius virens*) | **<0.005** | 0.003  ± 0.108 | 0.001 x10^-1^ ± 0.008 | 0.04 |
| Redfish (>10 cm)  (*Sebastes fasciatus*) | **<0.005** | 0.423  ± 0.939 | 0.060  ± 0.275 | 0.54 |
| Redfish Swimming (>10 cm)  (*Sebastes fasciatus*) | **<0.005** | 0.259  ± 0.692 | 0.195 x10^-1^ ± 0.167 | 0.49 |
| Redfish Resting (>10 cm)  (*Sebastes fasciatus*) | **<0.005** | 0.164  ± 0.566 | 0.041  ± 0.214 | 0.30 |
| Redfish (<10 cm)  (*Sebastes fasciatus*) | **<0.005** | 0.193  ± 0.599 | 0.003  ± 0.052 | 0.46 |
| Urophycid hake (~20cm)  (*Urophycis chuss or tenuis*) | 0.205 | 0.346 x10^-1^  ± 0.187 | 0.322 x10^-1^ ± 0.183 | 0.01 |
| Urophycid hake (<10cm)  (*Urophycis chuss or tenuis*) | **<0.005** | 0.016  ± 0.127 | 0.003  ± 0.058 | 0.13 |
| Silver Hake (*Merluccius bilinearis*) | **<0.005** | 0.724 x10^-1^  ± 0.302 | 0.614 x10^-2^ ± 0.081 | 0.31 |
| Snakeblenny  (*Lumpenus lampretaeformis*) | 0.062 | 0.002 x10^-1^ ± 0.016 | 0 | 0.02 |
| Witch Flounder  (*Glyptocephalus cynoglossus*) | 0.282 | 0.008 x10^-2^ ± 0.009 | 0 | 0.01 |

Table 8 Seasonal patterns, results from Kruskall Wallis test, the average +- stdev of the abundances and the Cohen’s d values

|  | Seasonal (p-value) | Summer | Autumn | Winter | Spring |
| --- | --- | --- | --- | --- | --- |
| American Plaice  (*Hippoglossoides platessoides*) | **0.03** | 0.003  ± 0.003 | 0.004  ± 0.060 | 0.002  ± 0.042 | 0.002  ± 0.041 |
| Atlantic Cod  (*Gadus morhua*) | 0.04 | 0 | 0.007x10^-1^  ± 0.026 | 0 | 0.002x10^-1^ ± 0.012 |
| Striped Atlantic Wolffish  (*Anarhichas lupus*) | 0.66 | 0 | 0.001x10^-1^  ± 0.010 | 0 | 0 |
| Blackbelly Rosefish  (*Helicolenus dactylopterus*) | 0.12 | 0 | 0 | 0.001x10^-1^ ± 0.012 | 0.005x10^-1^  ± 0.022 |
| Buckler Dory  (*Zenopsis conchifer*) | 0.67 | 0 | 0 | 0.001x10^-1^ ± 0.012 | 0.001x10^-1^ ± 0.012 |
| Fourbeard Rockling  (*Enchelyopus cimbrius*) | **0.012** | 0 | 0.001x10^-1^ ± 0.010 | 0.001x10^-1^ ± 0.011 | 0.001  ± 0.030 |
| Girard’s Hagfish  (*Myxine glutinosa?)* | 0.367 | 0 | 0.002x10^-1^ ± 0.014 | 0 | 0 |
| Haddock  (*Melanogrammus aeglefinus*) | 0.663 | 0 | 0.001x10^-1^ ± 0.010 | 0 | 0 |
| Longhorn Sculpin  (*Myoxocephalus octodecemspinosus*) | **<0.005** | 0.003  ± 0.053 | 0.001x10^-1^  ± 0.011 | 0.001x10^-1^  ± 0.011 | 0.012x10^-1^  ± 0.030 |
| Monkfish  (*Lophius americanus*) | 0.84 | 0 | 0.001x10^-1^ ± 0.011 | 0.001x10^-1^ ± 0.011 | 0 |
| Ocean Pout  (*Zoarces americanus*) | 0.03 | 0 | 0.001x10^-1^ ± 0.011 | 0.002x10^-1^ ± 0.015 | 0.001  ± 0.030 |
| Pollock  (*Pollachius virens*) | **<0.005** | 0.010  ± 0.100 | 0.002  ± 0.080 | 0.002x10^-1^ ± 0.015 | 0.002  ± 0.100 |
| Redfish (>10 cm)  (*Sebastes fasciatus*) | **<0.005** | 0.824 x10^-1^  ± 0.413 | 0.117  ± 0.408 | 0.293  ± 0.807 | 0.344  ± 0.874 |
| Redfish Swimming (>10 cm)  (*Sebastes fasciatus*) | **<0.005** | 0.630 x10^-1^ ± 0.327 | 0.879x10^-1^ ± 0.363 | 0.160  ± 0.563 | 0.172  ± 0.608 |
| Redfish Resting (>10cm)  (*Sebastes fasciatus*) | **<0.005** | 0.019  ± 0.179 | 0.029  ± 0.180 | 0.133  ± 0.497 | 0.172  ± 0.565 |
| Redfish (<10 cm)  (*Sebastes fasciatus*) | **<0.005** | NA | 0.105  ± 0.359 | 0.161  ± 0.526 | 0.337 ± 0.815 |
| Urophycid hake (~20 cm)  (*Urophycis chuss or tenuis*) | **<0.005** | 0.176 x10^-1^ ± 0.138 | 0.261x10^-1^ ± 0.165 | 0.328x10^-2^  ± 0.183 | 0.479x10^-1^  ± 0.218 |
| Urophycid hake (<10 cm)  (*Urophycis chuss or tenuis*) | **<0.005** | NA | 0.013  ± 0.115 | 0.012  ± 0.109 | 0.137x10^-2^ ± 0.037 |
| Silver Hake (*Merluccius bilinearis*) | **<0.005** | 0.833x10^-2^ ± 0.091 | 0.346x10^-1^ ± 0.212 | 0.238x10^-1^ ± 0.163 | 0.622x10^-1^ ± 0.286 |
| Snakeblenny  (*Lumpenus lampretaeformis*) | 0.576 | 0 | 0.001x10^-1^ ± 0.01 | 0.002x10^-1^ ± 0.015 | 0 |
| Witch Flounder  (*Glyptocephalus cynoglossus*) | **<0.005** | 0.009x10^-1^ ± 0.030 | 0 | 0 | 0 |

Table 9 Overview of all lander’s location, depth, deployment and recovery dates

|  |  | **Location** | | **Depth (m)** | **Dates** | |
| --- | --- | --- | --- | --- | --- | --- |
| **Site no.** | **Lander deployment no.** | **Latitude** | **Longitude** |  | **Placement** | **Recovery** |
| LS1 | M2201 | 43⁰ 53.2ˈ N | 63⁰ 05.7ˈ W | 152.33 | 11 Sep. 2021 | 12 May 2022 |
| LS2 | M2202 | 43⁰ 53.7ˈ N | 63⁰ 04.7ˈ W | 151.86 | 12 Sep. 2021 | 12 May 2022 |
| LS3 | M2179 | 43⁰ 54.8 N | 63⁰ 03.4 ˈ W | 221.87 | 12 Sep. 2021 | 12 May 2022 |
| LS1 | M2263 | 43°53.2’N | 63°05.9’W | 156.00 | 7 Oct. 2022 | 29 July 2023 |

Table 10 Overview of which lander contained what data: Conductivity, temperature, and depth (CTD), Infinity Current Meter (Inf.), Acoustic Doppler Current Profiler (ADCP), Sediment Trap (Sed. Trap), Chlorophyll a (Chl a), Passive Acoustic Monitoring devices, such as SoundTraps and AMARs (PAM).

| Benthic lander | CTD | Inf. | ADCP | Sed. Trap | Chl *a* | PAM |
| --- | --- | --- | --- | --- | --- | --- |
| LS1 (M2201) | √ | x | √ | √ | √ | √ |
| LS2 (M2202) | √ | x | √ | √ | √ | √ |
| LS3 (M2179) | √ | √ | x | x | √ | √ |
| LS1 (M2263) | √ | x | √ | √ | √ | √ |

Figure 3 Lander technical details. Lander’s view area measurements


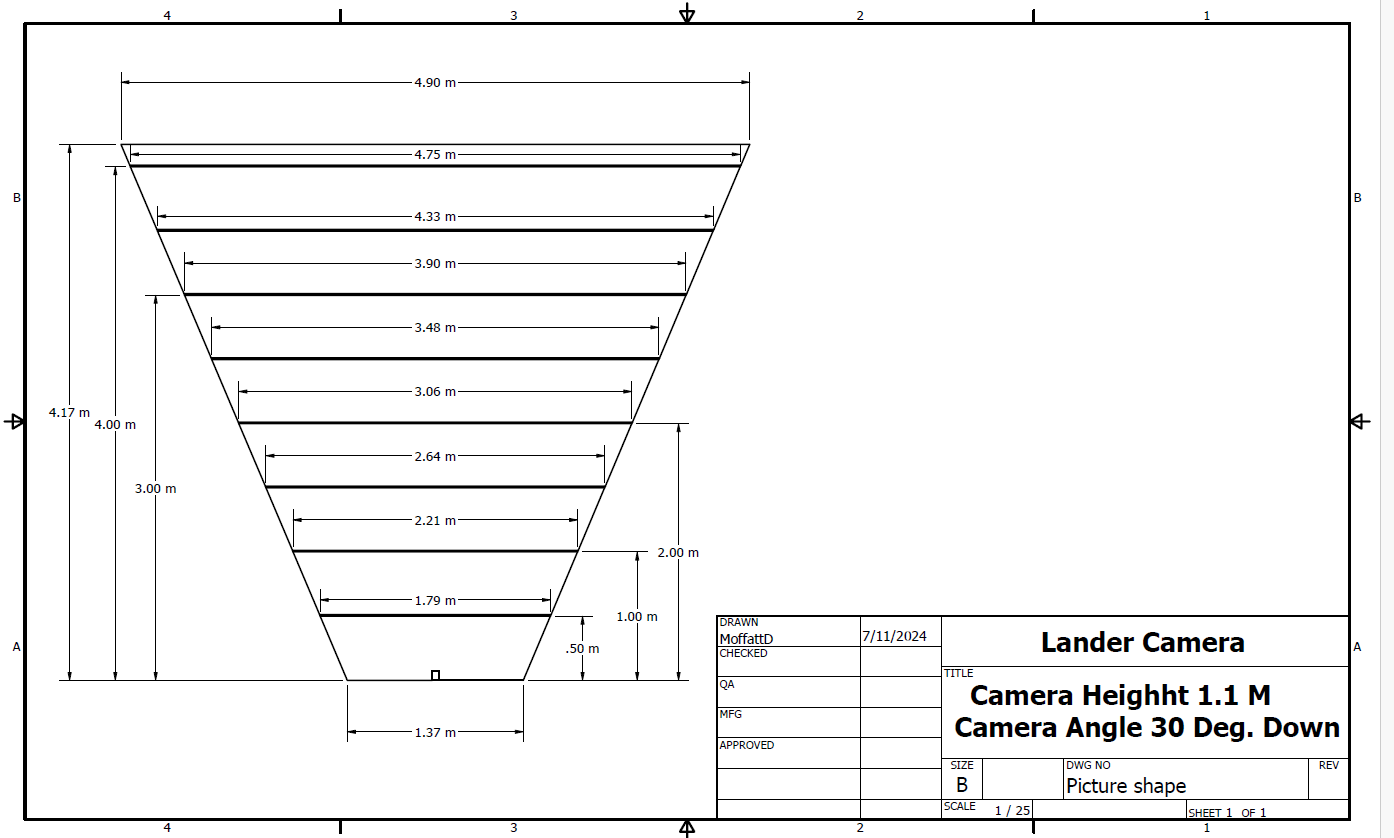


Figure 4 Time-lapse camera – diagram of custom set up


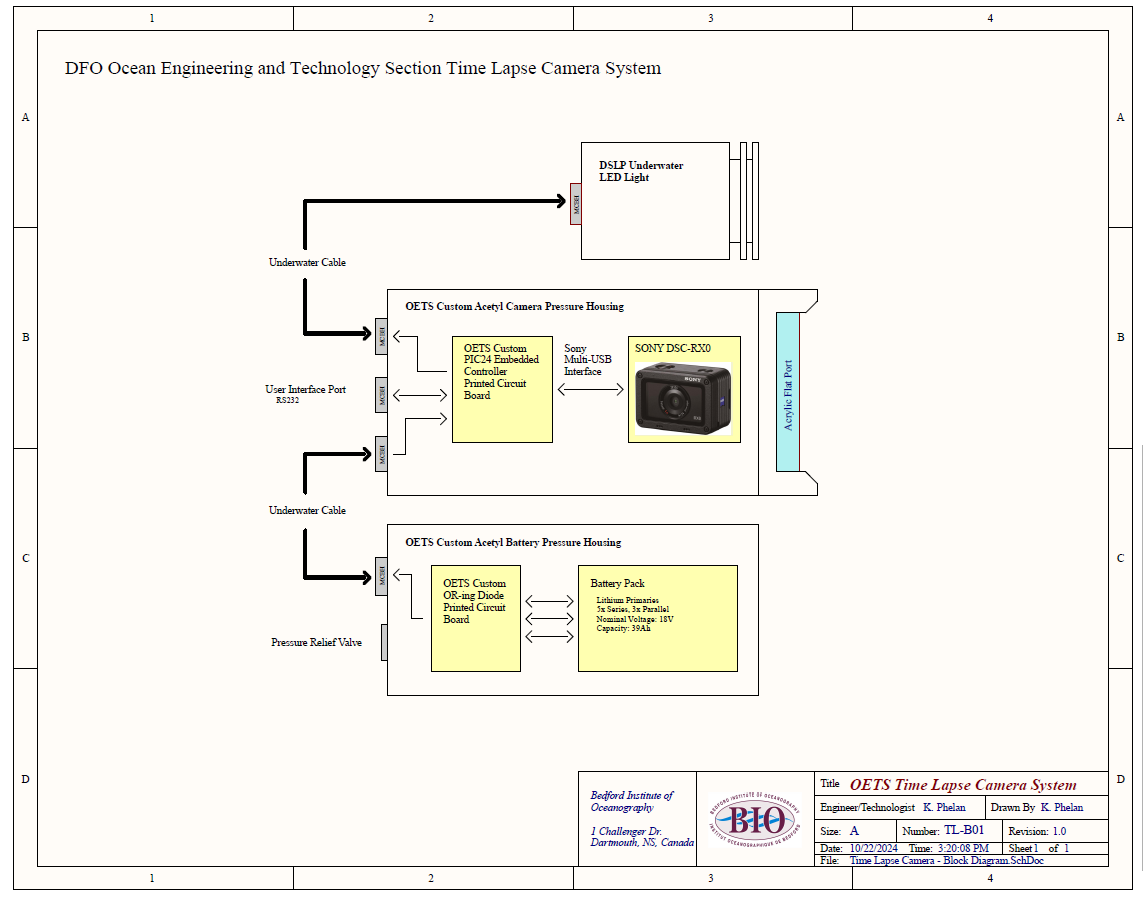

Supplement: Supplementary file 1 — Supplementary Information. [file 41598_2025_1822_MOESM1_ESM.docx]
